# Mouse models of radiation-induced glioblastoma

**DOI:** 10.18632/oncoscience.278

**Published:** 2015-12-28

**Authors:** Bipasha Mukherjee, Pavlina K. Todorova, Sandeep Burma

**Affiliations:** Division of Molecular Radiation Biology, Department of Radiation Oncology, University of Texas Southwestern Medical Center, Dallas, TX, USA

**Keywords:** glioma, Met, DNA double-strand break, ionizing radiation, particle therapy

Glioblastomas (GBM) are lethal brain tumors that can be triggered by exposure to ionizing radiation (IR), even at low doses from CT scans [1]. High doses of IR are also used to treat GBM, but the irradiated tumors inevitably recur. This raises the possibility that genomic changes induced by radiation may contribute not only to glioma initiation, but also to tumor recurrence. Thus, there is a compelling need for experimental model systems that recapitulate the process of radiation-induced gliomagenesis. Such models could not only help predict GBM-development risks from radiation exposure, but also help identify genetic alterations defining radiation-induced GBM, thereby facilitating the development of rational therapies for treating these recalcitrant tumors.

Our study published in the journal Oncogene employed a systematic approach to develop sensitive mouse models that can be used to study radiation-induced gliomagenesis [2]. *Ink4a, Ink4b* and *Arf* are key tumor suppressor genes that are deleted in a majority of GBMs [3]. We utilized transgenic mice with brain-restricted deletions of these tumor suppressors, individually and in combination, and examined their susceptibility to IR-induced GBM development. The most deleterious lesion inflicted by IR is the DNA double-strand break (DSB). We have shown previously that accelerated ions (particle radiation) induce complex DSBs that are refractory to repair unlike the simple breaks induced by X-rays (electromagnetic radiation) which are repaired to completion [4]. Therefore, we intra-cranially irradiated these transgenic mice with either X-rays or accelerated Fe ions to understand the process of radiation-induced gliomagenesis, and how this may be influenced by DNA damage complexity. We found that these mice did not develop gliomas spontaneously, but were prone to GBM development after exposure to a single, moderate dose of radiation. Remarkably, we found that Fe ions were at least four-fold more effective than X-rays in inducing these tumors, thereby confirming that complex DSBs triggered by accelerated ions are more harmful than simpler breaks induced by X-rays. This finding has important implications as the use of particle radiation (such as protons and carbon ions) for cancer therapy is steadily increasing. Our work indicates that particle radiation could indeed turn out to be more effective than X-rays for tumor control, but this also raises the specter of increased likelihood of secondary cancers triggered by such radiation.

Interestingly, while wild type mice did not develop gliomas upon radiation exposure, loss of *Ink4a* and *Arf* was sufficient to render these mice susceptible to IR-induced gliomas; additional loss of *Ink4b* significantly increased tumor incidence. These observations indicate that Ink4a, Ink4b and Arf act as key barriers to radiation-induced gliomagenesis, and confirms previous results from our laboratory and others implicating Ink4b as an important “backup” tumor suppressor for Ink4a [5]. One of the most interesting findings of our study came from multimodal analyses of the IR-induced tumors and neurosphere cultures derived thereof. We found amplification of the receptor tyrosine kinase Met to be the most prominent oncogenic alteration in these tumors. Met amplification was critical for transformation as well as for the maintenance of a cancer stem cell phenotype via upregulation of the re-programming transcription factor Sox2. Recent studies of other cancers show that MET amplification enables cancer cells to evolve and survive under therapeutic pressure, and that MET amplification confers radioresistance to cancer cells [6]. In light of these studies and our results, we speculate that radiotherapy of GBM could engender clones of MET-amplified cancer cells that drive tumor recurrence. If so, recurrent glioblastomas may be particularly vulnerable to radiosensitization strategies involving the use of MET inhibitors.

We are currently validating additional transgenic models with brain-targeted deletions of other GBM-relevant tumor suppressor genes like *p53* and *Pten*. The use of distinct yet complementary mouse models could prove to be very useful for analyzing the mechanistic underpinnings of radiation-induced gliomagenesis. For example, by crossing these mice with DNA repair-deficient mouse models, we hope to understand how specific DSB repair pathways act as barriers to gliomagenesis. In the future, this study could also have “far”-reaching implications for astronauts 100 million miles away on the surface of Mars. These models (along with models of other cancers) are being used in NASA-funded studies to understand cancer risks for Mars-bound astronauts from space radiation which consists of accelerated ions such as the Fe ions used in this study [7]. In sum, validation of these sensitive yet simple mouse models sets the stage for in-depth mechanistic studies of radiation-induced gliomagenesis which could lead to effective approaches for treating radiogenic cancers.
